# Level of education, but not occupation, is differentially associated with asthma phenotypes in adults

**DOI:** 10.1002/clt2.12389

**Published:** 2024-08-13

**Authors:** Muwada Bashir Awad Bashir, Rani Basna, Göran Wennergren, Madeleine Rådinger, Helena Backman, Emma Goksör, Jan Lötvall, Linda Ekerljung, Hannu Kankaanranta, Bright I. Nwaru

**Affiliations:** ^1^ Krefting Research Centre Institute of Medicine Sahlgrenska Academy University of Gothenburg Gothenburg Sweden; ^2^ Division of Geriatric Medicine Department of Clinical Sciences in Malmö Lund University Lund Sweden; ^3^ Department of Pediatrics University of Gothenburg Gothenburg Sweden; ^4^ Department of Public Health and Clinical Medicine Section of Sustainable Health/the OLIN Unit Umeå University Umeå Sweden; ^5^ Department of Respiratory Medicine Seinäjoki Central Hospital Seinäjoki Finland; ^6^ Faculty of Medicine and Health Technology Tampere University Respiratory Research Group Tampere University Tampere Finland; ^7^ Wallenberg Centre for Molecular and Translational Medicine Institute of Medicine University of Gothenburg Gothenburg Sweden

## Abstract

Conclusion

Education, but not occupation, was differentially associated with adult asthma phenotypes in the general population. Further research into socioeconomic status variation in various asthma phenotypes is warranted.

To the Editor

Social gradients in asthma have been known for decades.^1^ However, evidence on the role of socioeconomic status (SES) in different asthma phenotypes is lacking. Asthma is a heterogeneous disease, characterized by variable clinical presentations, and influenced by physiological, severity, and demographic factors.^2^ Understanding SES variations across different asthma phenotypes can support SES‐based risk stratification, both for prevention and management.^3^

The ongoing longitudinal population‐representative West Sweden Asthma Study provides a unique setting to define asthma phenotypes in adults and to catalyze various clinical and epidemiological studies on asthma and respiratory health.^4^ By including 47 variables encompassing clinical, demographic, risk factor, asthma trigger, pulmonary function, and inflammation variables, we used a novel machine learning approach, deep learning clustering, to derive four clinically meaningful asthma phenotypes in adults (*n* = 1895). Full information on included variables and derived phenotypes can be found through this link. With clinical input, the derived phenotypes were descriptively named as follows:

‐ Phenotype 1 (*n* = 458, 24.2%): Troublesome late‐onset, non‐atopic asthma with smoking
‐ Phenotype 2 (*n* = 545, 28.7%): Female‐dominated early adult‐onset asthma
‐ Phenotype 3 (*n* = 358, 18.9%): Adult‐onset asthma with high inflammation
‐ Phenotype 4 (*n* = 534, 28.2%): Early onset, mild, atopic asthma

In the current paper, we assessed the variations in the frequency of the derived asthma phenotypes by SES, using education and occupation as indicators. Education was defined as the highest level attained by the subjects, categorized as primary, secondary, and tertiary education. Occupational classes were defined using the International Standard Classification of Occupations, categorized as highest, lower high, upper low, and lowest skill levels. We fitted a multinomial logistic regression model, one separately for each exposure, to study these associations, using the no asthma group (*n* = 1206) as the outcome reference group, adjusting for age, sex, body mass index, smoking status, occupational exposure to vapor, gas, or dust fumes, being raised on a farm, and urbanization level. The first categories of education (primary education) and occupation (lowest skill level) were used as the reference exposure categories. The estimates of the associations are presented as odds ratios (OR), accompanied by their 95% confidence intervals (95% CI).

Figure 1 presents the results of the logistic regression analysis. Those with secondary education (OR 1.61, 95% CI 1.26–2.07) were more likely to be in the first asthma phenotype, compared to being in the non‐asthma group, than those with primary education. Those with secondary (OR 0.44, 95% CI 0.29–0.66) and those with tertiary (OR 0.44, 95% CI 0.29–0.66) education were less likely to be in the second asthma phenotype (compared to being in the non‐asthma group) than those with primary education. Those with tertiary education (OR 0.76, 95% CI 0.59–0.97) were in addition less likely to be in the fourth asthma phenotype (compared to being in the non‐asthma group) than those with primary education. None of the educational levels was associated with the third asthma phenotype. Finally, occupation was not associated with any of the asthma phenotypes.

**FIGURE 1:**
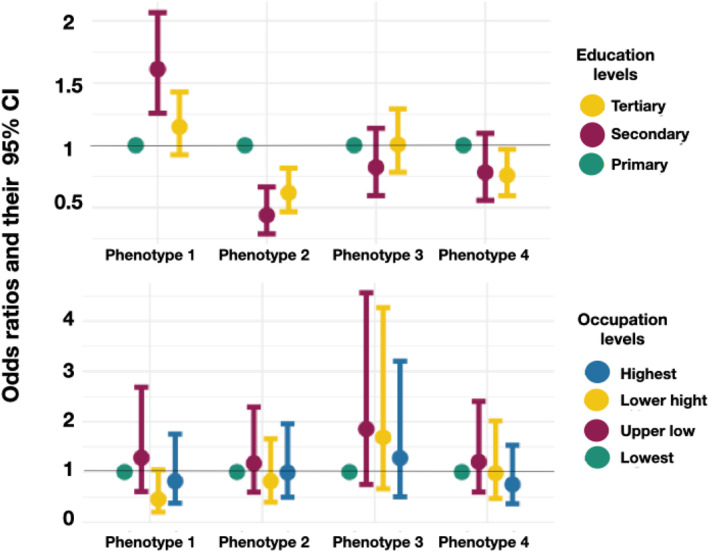
The association between education and occupation levels and risk of asthma phenotypes estimated as odd ratios and their 95% confidence intervals.

We found that levels of education were differentially associated with specific asthma phenotypes in adults. Our finding that showed less probability of female dominant mild asthma among those with secondary and tertiary education supports the notion that SES‐related impact on asthma likely is more pronounced in women than in men.^5^ The decreased odds of early onset, mild, atopic asthma in higher SES groups may stem from the potential for higher remission rate of this asthma phenotype than for other phenotypes in the context of reduced exposure to adverse environmental factors, which is commonly the characteristic of higher than lower SES groups.^6^ Our results also showed that SES may modify the inflammatory processes linked with our observed asthma phenotype in perhaps lesser degree than it does to the other phenotypes. Finally, none of the occupational classes were associated with any of the asthma phenotypes, which could stem from the fact that none of the asthma phenotypes was primarily occupational‐related, perhaps owing to the fact that we lacked detailed data on occupational exposures used for the clustering.^7^ However, we cannot exclude the possibility that certain factors or sensitizers are missed by the current occupational classification.

Our results can be applied to the adult population of western Sweden. The asthma phenotypes studied in the current study covered the spectrum of the main known asthma phenotypes, including aspects of severity and age of onset. Analyzing the role of SES in these phenotypes advances the investigations on SES variations in asthma. This study, to our knowledge represents the first attempt to define the role of SES in different asthma phenotypes in adults. Nevertheless, the cross‐sectional nature of our research limits the ability to address the temporal association between SES and the asthma phenotypes. Additionally, using more objective indicators of SES rather than education and occupation, which do not capture all aspects of social exposure, and addressing the healthy worker effect bias could provide a more accurate assessment.

In conclusion, our results indicate that education, rather than occupation, as an indicator of SES, was differentially associated with adult asthma phenotypes in the general population. The absence of any association with occupation could indicate an interplay of more complex sensitizers and irritants that are not readily captured by occupational classification. Further research into SES variation in various asthma phenotypes is warranted to complement the current findings. These studies can inform the development of tailored SES‐related prevention and management strategies for asthma.

## AUTHOR CONTRIBUTIONS

**Muwada Bashir Awad Bashir**: Investigation; methodology; visualization; software; formal analysis; data curation; writing – original draft; writing – review & editing; validation. **Rani Basna**: Conceptualization; methodology; writing – review & editing. **Göran Wennergren**: Conceptualization; funding acquisition; writing – review & editing. **Madeleine Rådinger**: Conceptualization; funding acquisition; resources; writing – review & editing. **Helena Backman**: Conceptualization; funding acquisition; writing – review & editing; resources. **Emma Goksör**: Conceptualization; funding acquisition; supervision. **Jan Lötvall**: Conceptualization; funding acquisition. **Linda Ekerljung**: Conceptualization; funding acquisition; supervision; writing – review & editing. **Hannu Kankaanranta**: Conceptualization; funding acquisition; supervision; writing – review & editing. **Bright I. Nwaru**: Conceptualization; funding acquisition; supervision; writing – review & editing; resources.

## CONFLICT OF INTEREST STATEMENT

HK reports personal fees for lectures and consulting from AstraZeneca, Boehringer‐Ingelheim, Chiesi, COVIS Pharma, GSK, Medscape, MSD, Novartis, Orion Pharma and Sanofi. All other authors of this work declare no conflict of interest related to current work.
